# Combined three-part humeral anterior fracture-dislocation and humeral shaft fracture treated with one-stage long stem shoulder hemiarthroplasty in an active elderly patient: A case report and review of literature

**DOI:** 10.1051/sicotj/2017045

**Published:** 2017-10-30

**Authors:** Guillaume Herzberg, Eloise Tebaa

**Affiliations:** 1 Department of Orthopaedic Surgery, Shoulder and Elbow Surgery Unit, Herriot Hospital 69003 Lyon France

**Keywords:** Spiral humeral shaft fracture, Three-part humeral head fracture, Three-part humeral head fracture-dislocation, Hemiarthroplasty, Long stem hemiarthroplasty

## Abstract

*Introduction*: Injuries combining a humeral head fracture-dislocation and a shaft fracture of the ipsilateral humerus are very rare. They should be separated from extended fractures of the humeral head to the shaft [1].

*Case report*: We present the case of an active 84-year-old man who sustained a three-part fracture-dislocation of the proximal humerus combined with a long spiral humeral middle third diaphyseal fracture, after a ski fall. We were unable to find a similar case in the literature. He was treated with a long stem hemiarthroplasty, associated with screw osteosynthesis of the long spiral shaft fracture. The result after 30 months of follow-up was excellent, with good shoulder range of motion, good bone integration of the prosthesis and uneventful healing of the fracture.

*Conclusion*: This treatment allowed this intrepid elderly patient to recover a normal quality of life, including driving his car and to return to skiing.

## Introduction

Proximal humeral fractures are common injuries, especially in the elderly with osteoporotic bone. However, combined fractures of the proximal humerus and its ipsilateral shaft are highly uncommon. These injuries occurred most often as a result of high energy trauma in young or middle-aged patients. As the literature concerning this rare injury is limited to case reports where various treatments were applied, there is currently no consensus management.

The purpose of this paper is to report the management and 30 months’ follow-up clinical result of an 84-year-old healthy retired surgeon who presented with this unusual traumatic fracture after a ski fall. He was treated at the acute stage with a long stem humeral hemiarthroplasty combined with screw fixation of the humeral shaft. We also discuss alternative treatment recommendations from the literature.

## Case report

A very active 84-year-old male retired general surgeon fell while skiing.

**Figure 1.**
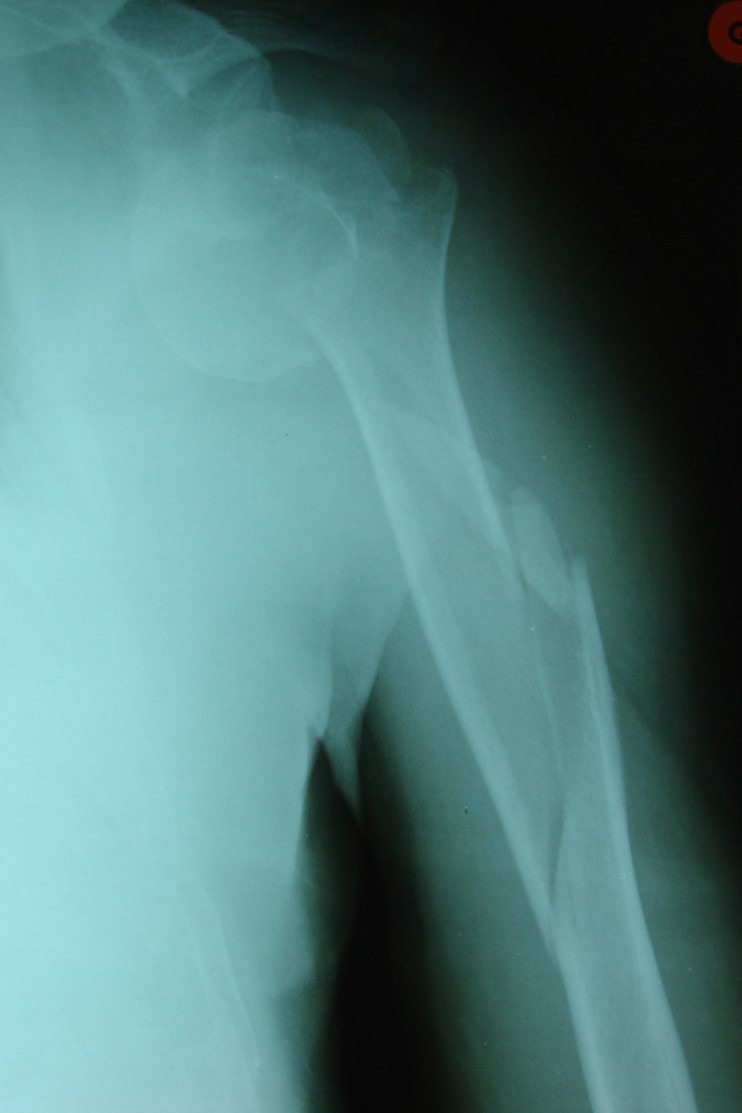
Initial X-ray of our patient.

He sustained a left closed injury combining a three-part anterior fracture-dislocation of the humeral head and an ipsilateral long spiral displaced humeral shaft fracture (Figure 1). There were no vascular or neurological complications.

**Figure 2.**
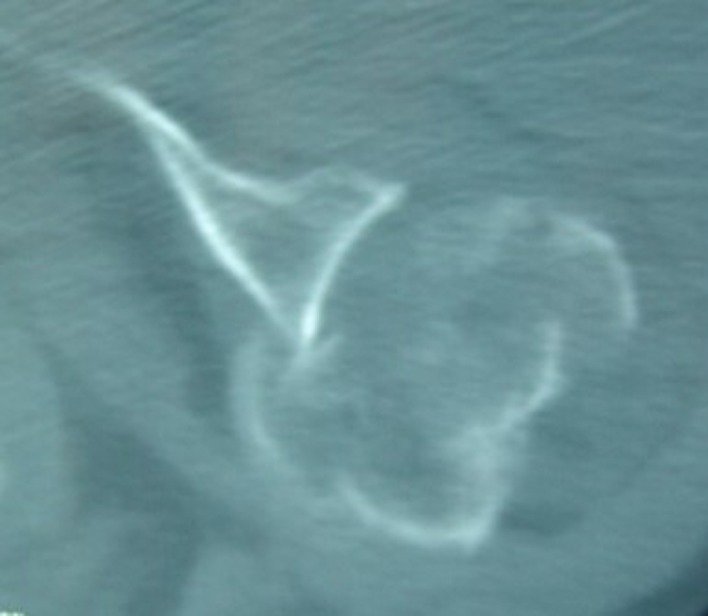
Initial CT scan.

He was referred to our unit after a failed attempt at closed reduction in the local hospital. We performed a computed tomography (CT) scan (Figure 2). The senior author of this paper (GH) treated both fractures in a single-stage operation, using a long stem humeral hemiarthroplasty combined with screw fixation of the long spiral humeral shaft fracture. The operation was performed in a beach chair position under general anesthesia and interscalene block.

A distally extended deltopectoral approach was used to expose both fractures. At the proximal humerus, the tuberosities with their attached rotator cuff tendons were separated from the anteriorly dislocated head, which was removed. A 1 cm cuff tear at the junction of supra and infraspinatus tendons was found. The intra-articular part of the long head of biceps tendon showed fraying, widening and degenerative changes.

A shoulder hemiarthroplasty with a 200 mm stem in 30° of retroversion was performed. The stem was cementless in the proximal part of humeral shaft and cemented in the distal part. A trial implant introduced from proximal to distal into the two fragments was used to determine the optimal height of the implant. The tension of the trial humeral head implant on the long head of the biceps and the distance between the top of the prosthetic head and the upper border of the pectoralis major tendon [2] were also used to optimize the height of the humeral implant.

After removal of the intra-articular part of the long head of the biceps tendon, the final implant was introduced into the proximal humeral fragment using an appropriate 30° retroversion (with reference from the forearm) and cemented into the reduced distal fragment using reduction forceps. The spiral humeral shaft fracture was anatomically reduced around the stem of the definitive implant and fixed with four cortical screws.

The lesser and greater tuberosities were then reduced and fixed in anatomical position on the prosthesis with non-absorbable sutures. Cancellous bone grafting was used to fill in the bone loss between the greater tuberosity and the implant. The small supraspinatus cuff tear was reinserted into the reduced and fixed greater tuberosity using transosseous sutures. A tenodesis of the long head of the biceps tendon was performed in its groove at the upper border of the pectoralis major using non-absorbable sutures.

**Figure 3.**
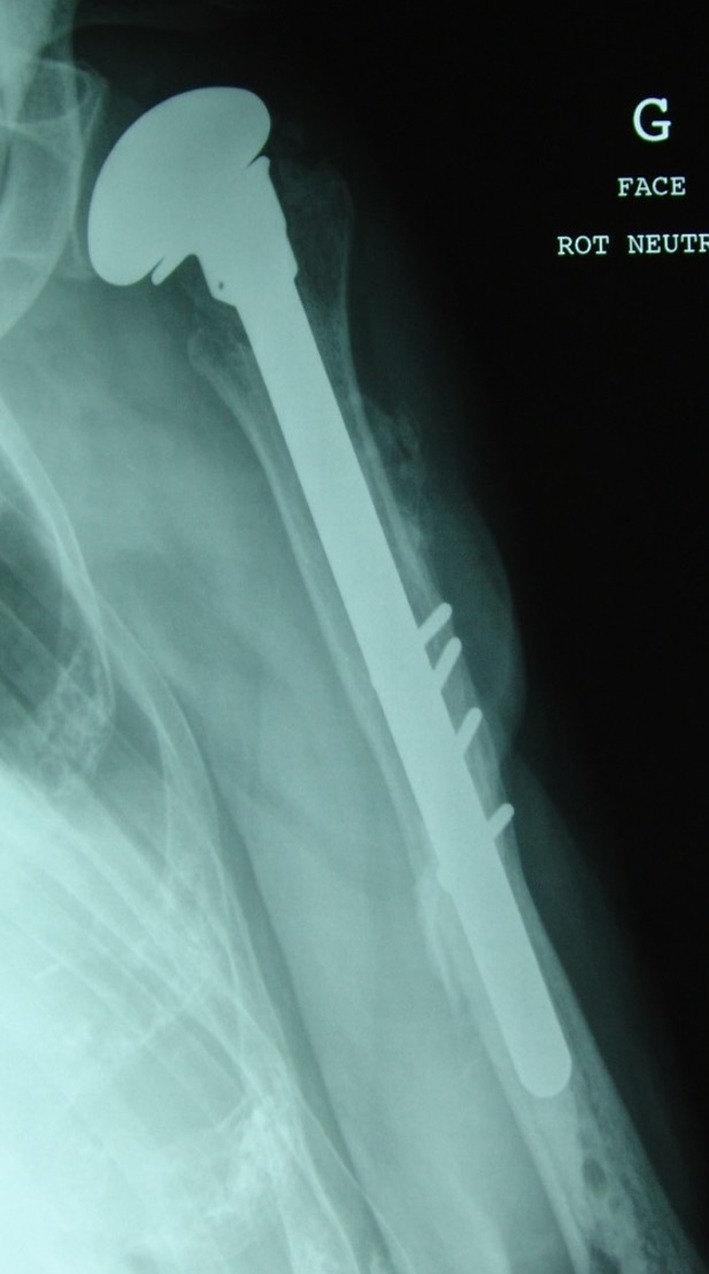
Immediate postoperative X-ray showing anatomical reduction of the fractures and good position of the prosthesis.

Postoperative radiographs showed anatomical reduction of the fractures and good position of the prosthesis (Figure 3).

Postoperatively the arm was placed into a standard shoulder sling for six weeks. Postoperative management consisted of early rehabilitation with pendulum passive exercises at day 1, with the addition of passive controlled external rotation exercises three weeks later. Uneventful healing of the tuberosities to the implant as well as the humeral shaft fracture were observed at eighth week of follow-up.

**Figure 4.**
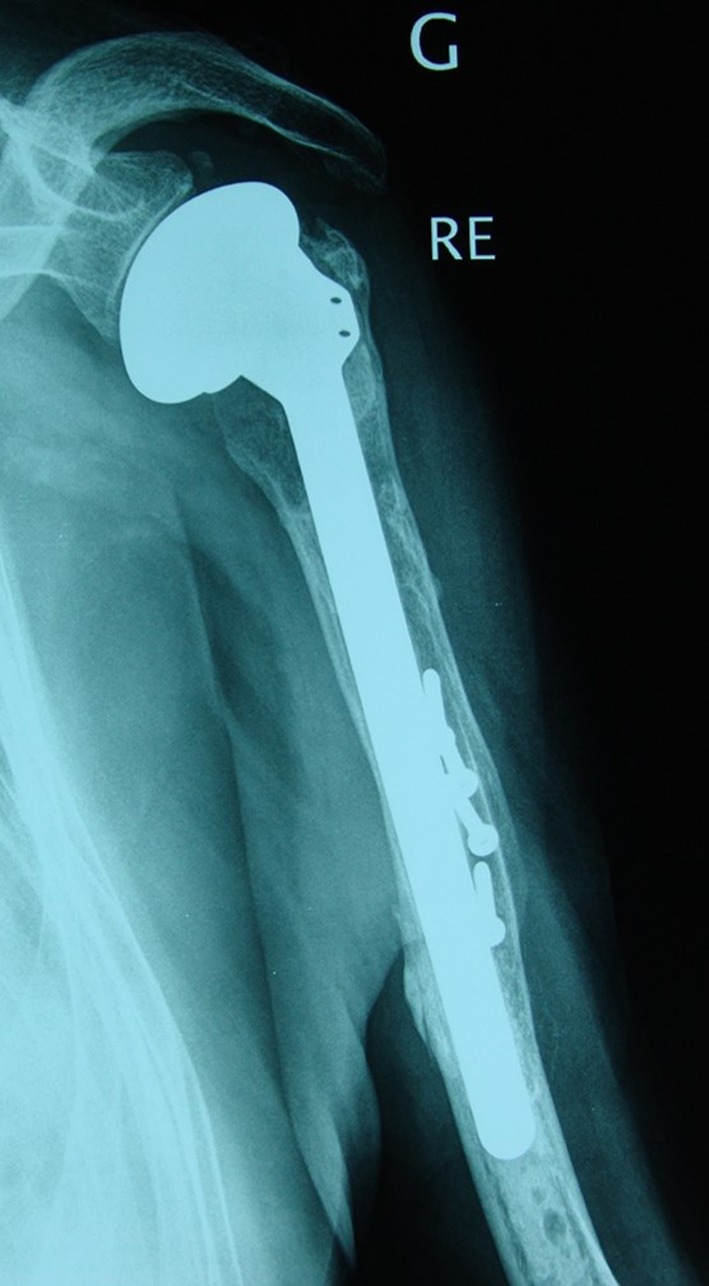
X-rays at 30 months follow-up confirmed satisfactory healing of both the tuberosities (without osteolysis) and of the humeral shaft fracture. No signs of stem or implant loosening were observed.

The patient was reviewed by an independent observer at 30 months’ follow-up. His left shoulder and arm were pain free. Active and passive elevation as well as abduction reached 140°, external rotation 35°, and internal rotation reached the fourth lumbar vertebrae. The absolute Constant score was 67% (Ponderated Constant score was 100%). The patient was able to perform his daily activities including driving his car without pain. The 30 months’ follow-up radiographs confirmed satisfactory healing of both the tuberosities (without osteolysis) and the humeral shaft. No signs of stem or implant loosening were observed (Figure 4).

## Discussion

We report a case of an acute three-part anterior fracture-dislocation of the proximal humerus combined with an ipsilateral spiral humeral mid shaft fracture in an elderly male treated with a one-stage long stem hemiarthroplasty combined with screw fixation of the humeral shaft fracture. The clinical and radiological results were good at 30 months’ follow-up. Our patient was aged, retired general surgeon, yet very active, with important functional needs including sports (ski). This confirms that a good result may be obtained with a hemiarthroplasty for three-part humeral fracture in an elderly, provided that care is taken to meticulous reinsertion of the humeral tuberosities [3].

Fracture-dislocation of the shoulder combined with ipsilateral humeral shaft fracture is an extremely rare entity. “Combined” humeral head and shaft fractures (with an intact bony segment in between the two fractures) are different from humeral head fractures “extended” distally to the shaft [1].

Only a few cases of combined humeral head and shaft fractures have been reported in the literature. Previous publications involved mostly young patients, the injuries being caused by high energy traffic accidents involving motorcycles or cars, falls from heights or workplace machinery accidents.

Most of the cases were two-part fracture-dislocations of the greater tuberosity or the surgical neck combined with humeral shaft fracture. To the best of our knowledge there are only anecdotal reports about three-part or four-part proximal humeral fractures combined with ipsilateral humeral shaft fractures.

Yang [4] reported on eight cases of three-part humeral head fractures with concomitant humeral shaft fractures. Only two cases had “combined” fractures of the head and shaft according to the above definition. All others were three-part fractures “extended” to the proximal shaft. There were no humeral fracture-dislocations in this series. The average age of this series was 50 years (min. 24–max. 61 years). All patients were treated with long helical plates, with good results except in two cases where abutment of the plate on the acromion occurred, and two cases where production of abundant callus around the plate prevented removal.

Long plate fixation would have been doomed to failure in our 84-year-old patient since plate fixation in elderly osteoporotic three-part fractures is unreliable. Most authors currently recommend treatment of three- and four-part humeral head fractures in the elderly with arthroplasty. Acceptable results and low revision rates can be obtained, provided that tuberosities healing occurs.

Flint [5] reported on one case of three-part humeral head fracture combined with ipsilateral humeral shaft fracture treated non-operatively a 69-year-old woman who refused surgery. There was no humeral head dislocation. As expected, non-operative treatment resulted in malunion at both fracture sites and poor clinical result at follow-up.

Garvanos [1] reported on six cases of combined humeral head (five 2-part and one 4-part) and diaphyseal fractures. Three cases were complicated by anterior shoulder dislocations that were reduced immediately after the injury. Standard antegrade nailing was performed in all cases. Only the four-part fracture combined with humeral shaft fracture in a 68-year-old woman was similar to our case. Fracture healing was obtained at the final 14 months’ follow-up. Shoulder flexion was 130°, external rotation 20° and the Constant score was 70%. However, pain was still present.

Garofalo [6] reported on a series of 22 patients aged 65 years or older presenting with humeral head fractures (three-part, four-part or two-part humeral head splitting) extended to the diaphysis. All patients were treated with reverse shoulder arthroplasty (RSA) and cerclage fixation. Our case was different in that it was the “combined” type of fracture, with an intermediate fragment between the humeral head fracture and the diaphyseal fracture. However, they validated the use of a long stem humeral implant for these complex injuries. We could have used a long stem reverse arthroplasty RSA for our patient but only short stem RSA was available at that time.

Perez Cervera [7] reported on a 63-year-old female who presented with an injury very similar to our patient’s but their patient was much younger. She sustained a three-part anterior fracture-dislocation combined with a spiral shaft fracture. There was an associated injury of the axillary artery, which was successfully treated in emergency by vascular surgeons. She was treated with standard stem length hemiarthroplasty, associated with osteosynthesis of the shaft fracture by a plate. The clinical result was not reported.

## Conclusion

To the best of our knowledge, no case equivalent to our case (i.e. “combined” humeral head fracture and shaft fracture in an 84-year-old male) has been reported in the literature. Nowadays if a shoulder surgeon encounters a similar case in an 84-year-old patient, he would probably choose a long stem RSA and shaft osteosynthesis. Our case is very interesting because it demonstrated that a long stem hemiarthroplasty with repair of a small cuff tear could provide a good outcome even in an elderly patient. The follow-up balance between external rotation and internal rotation in our patient would probably have been worse after RSA compared with hemiarthroplasty. Further case reports are necessary to improve our knowledge about these rare injuries when they occur in the elderly.

## Conflict of interest

The authors declare that they have no conflict of interests.
